# Myocardial ischemia–reperfusion injury after acute myocardial infarction: spatiotemporal mechanisms and endotype-matched multi-target modulation by compounds from traditional Chinese medicine

**DOI:** 10.3389/fcvm.2026.1797330

**Published:** 2026-04-15

**Authors:** Lin Sun, Ruiqian Guan, Ming Gao, Zhaoyang Gu, Miao Zhang

**Affiliations:** 1 Department of Acupuncture, The Second Affiliated Hospital of Heilongjiang University of Chinese Medicine, Harbin, Heilongjiang, China; 2 Department of Tuina, The Second Affiliated Hospital of Heilongjiang University of Chinese Medicine, Harbin, Heilongjiang, China; 3 Department of Cardiology, The First Affiliated Hospital of Harbin Medical University, Harbin, Heilongjiang, China

**Keywords:** acute myocardial infarction, endotypes, immunothrombosis, intramyocardial hemorrhage, ischemia–reperfusion injury, microvascular obstruction, traditional Chinese medicine compounds

## Abstract

Myocardial ischemia–reperfusion (I/R) injury remains a major determinant of outcome after acute myocardial infarction (AMI) despite timely primary percutaneous coronary intervention (PPCI). Although epicardial patency is routinely restored, incomplete tissue reperfusion, microvascular obstruction (MVO), intramyocardial hemorrhage (IMH), and maladaptive inflammation often limit myocardial salvage and promote adverse remodeling. In this narrative review, I/R injury is synthesized as a spatiotemporal, multicompartment network in which dominant mechanisms and actionable nodes shift from seconds to weeks and across cardiomyocytes, the coronary microvasculature, and systemic immune–metabolic programs. This framework helps explain why many single-target, single-window cardioprotective strategies have shown robust preclinical benefit yet failed to improve outcomes in contemporary ST-segment elevation myocardial infarction (STEMI) trials, where heterogeneity in ischemic time, comorbidities, microvascular injury patterns, and background pharmacotherapy reshapes injury topology and dilutes target-dominant subgroups. We propose an endpoint-anchored approach that distinguishes cardiomyocyte-vulnerability, microvascular-bottleneck, and resolution-deficient endotypes and matches interventions to phase-specific objectives using compartment-aligned endpoint stacks and target-engagement biomarkers. Within this rationale, defined bioactive compounds derived from traditional Chinese medicine (TCM) and selected standardized preparations are evaluated as potential network modulators with plausible actions on mitochondrial vulnerability, immunothrombosis, endothelial integrity, and inflammatory resolution. Translational priorities include stringent standardization, pharmacokinetic/pharmacodynamic (PK/PD)-informed dosing, PCI-compatible safety and drug–drug interaction assessment, and biomarker-guided trial designs enriched for mechanistically matched endotypes.

## 1 Introduction

Acute myocardial infarction (AMI), particularly ST-segment elevation myocardial infarction (STEMI), continues to impose substantial mortality and long-term disability despite mature reperfusion systems (1). Primary percutaneous coronary intervention (PPCI) restores epicardial patency in most patients with STEMI, but successful reopening of the culprit artery does not guarantee complete tissue reperfusion. In this setting, myocardial ischemia–reperfusion (I/R) injury refers specifically to the additional myocardial and microvascular damage triggered or amplified by reperfusion itself, superimposed on the antecedent ischemic insult. Abrupt metabolic, ionic, and inflammatory transitions at reperfusion can amplify cardiomyocyte loss, disrupt microvascular perfusion, and impair infarct healing (2).

Clinically, the paradox of restored epicardial flow without fully rescued myocardium is most clearly reflected by tissue-level reperfusion failure. This includes microvascular-domain phenotypes such as no-reflow, microvascular obstruction (MVO), and intramyocardial hemorrhage (IMH), together with their downstream consequences for cardiomyocyte survival, infarct healing, and ventricular remodeling (3). These phenomena can persist despite angiographically successful PPCI and are consistently associated with worse remodeling trajectories and higher risk of heart failure. In practice, the persistence and spatial distribution of microvascular injury often determine whether early salvage translates into durable functional recovery.

Despite decades of intensive research, therapies that consistently reduce I/R injury in patients remain elusive (4). Numerous cardioprotective strategies have delivered robust benefit in experimental settings but failed to improve outcomes in large clinical trials (5). These repeated neutral results point to a common design–biology mismatch: many interventions were optimized to inhibit a single pathway or target a narrow time window, whereas human I/R injury is shaped by ischemic duration, comorbidities, background pharmacotherapy, and heterogeneous microvascular and immune responses. A more useful conceptualization is that myocardial I/R injury behaves as a spatiotemporal, multicompartment network in which dominant mechanisms and actionable nodes shift over minutes to weeks and across anatomical and systemic compartments.

Within this network, at least three interacting compartments can be delineated. In cardiomyocytes, early reperfusion triggers oxidative bursts, calcium overload, and mitochondrial dysfunction that converge on regulated cell death programs and bioenergetic failure (6). Beyond apoptosis, necroptosis, pyroptosis, and ferroptosis, integrative frameworks such as PANoptosis highlight coordinated crosstalk among death pathways (7). Emerging metabolic death concepts (including copper-dependent proposals discussed in cuproptosis-related studies) have also entered cardiovascular discourse, but current evidence remains predominantly preclinical and often associative; their causal contribution and druggability in human reperfused myocardial infarction require direct validation (8). In the coronary microvasculature, endothelial barrier disruption and glycocalyx shedding can expose adhesion and procoagulant surfaces, facilitating leukocyte–platelet interactions and amplifying thrombo-inflammatory signaling (9). Neutrophil extracellular traps (NETs) and microthrombi can further promote immunothrombosis and contribute to no-reflow, making microvascular integrity a determinant of both acute salvage and downstream healing (10). In parallel, pericyte constriction and capillary compression may provide a structural basis for persistent microvascular hypoperfusion even after successful epicardial recanalization (11). Systemically, immune–metabolic reprogramming can amplify injury or promote resolution; lactate signaling and lactylation have been proposed as links between metabolic stress and macrophage phenotype, but causal inference in reperfused AMI remains limited and likely context-dependent (12).

To improve conceptual clarity, three operational terms are used throughout this review. First, an endotype refers to a mechanistically defined subgroup in which a particular injury bottleneck is relatively dominant, even though multiple pathways remain active. Second, an endpoint stack refers to a pre-specified, compartment-aligned combination of proximal, intermediate, and downstream readouts used together to infer whether a therapy has modified the intended network state. Third, target-engagement biomarkers are measurable indicators that an intervention has reached and perturbed its proposed biological node in humans. In this framework, target engagement supports mechanistic plausibility, whereas endpoint stacks help bridge early biological effects to later clinical consequences such as adverse remodeling or heart-failure risk.

This narrative review first anchors I/R mechanisms to clinically meaningful phenotypes and endpoints in reperfused myocardial infarction (13). It then maps the spatiotemporal injury network across cardiomyocytes, microvasculature, and systemic immunity–metabolism, highlighting actionable nodes alongside key uncertainties. Finally, evidence for representative TCM-derived compounds and selected standardized preparations is synthesized as potential network modulators, with explicit node mapping, evidence-level restraint, and a translational roadmap toward biomarker-guided precision cardioprotection (14, 15).

## 2 Clinical phenotypes and endpoints that anchor mechanisms

Mechanistic discussions of myocardial I/R injury can easily become pathway catalogs unless anchored to clinical phenotypes that are measurable, time-resolved, and causally informative. In reperfused AMI, the most useful endpoints are those that partition the injury network into interpretable states, for example separating cardiomyocyte-dominant loss from microvascular-dominant perfusion failure, and distinguishing inflammatory amplification from adaptive resolution. Accordingly, clinical readouts can be treated as network observables across three layers: myocardial salvage, microvascular integrity, and the transition from acute injury to remodeling (16).

### 2.1 Myocardial salvage as a multidimensional endpoint

Myocardial salvage should not be represented by a single summary metric, because necrosis, edema, microvascular injury, and early functional recovery evolve on different timelines and may respond differently to treatment. Infarct size remains an important endpoint, but on its own it cannot distinguish irreversible necrosis from concurrent edema, microvascular injury, or delayed tissue recovery, all of which follow different temporal trajectories. A more informative approach is to treat myocardial salvage as a set of partially orthogonal readouts: irreversible necrosis, transient edema/inflammation, and early contractile reserve (17). Mapping-based tissue characterization can capture the evolution of edema and inflammation over the first week, while deformation imaging provides a functional counterpart that is often more sensitive than ejection fraction early after PPCI (18). This separation is mechanistically valuable because therapies that stabilize mitochondria and limit regulated cell death would be expected to preferentially shift necrosis-related signals, whereas interventions that reshape immune–metabolic programs may more strongly influence edema-resolution trajectories and early strain recovery.

### 2.2 Microvascular injury defines a distinct endotype

Contemporary reperfusion science recognizes that epicardial patency is an imperfect surrogate for tissue reperfusion. MVO and IMH identify a microvascular endotype in which endothelial barrier failure, immunothrombosis, erythrocyte extravasation, and capillary compression can dominate the clinical course (19). Microvascular injury is therefore best treated as a graded and spatially distributed phenotype rather than a binary variable: severity, distribution (core vs. border-zone), and persistence over the first week imply different dominant mechanisms and therapeutic windows (20). Integrating invasive microcirculatory indices (such as IMR where available) with imaging-defined MVO/IMH can operationalize no-reflow biology into trial-ready endpoints and reduce mechanistic ambiguity (21).

### 2.3 Inflammation is a trajectory rather than “high vs. low”

The post-reperfusion immune response is increasingly understood as a sequence of state transitions rather than a single inflammatory peak. Conventional markers such as leukocyte count and C-reactive protein are coarse and can miss the clinically decisive feature: whether inflammation resolves appropriately. A more mechanistically aligned strategy is to measure trajectories that reflect immune–metabolic programming and resolution capacity, including time-dependent shifts in circulating myeloid phenotypes, signals related to damage-associated molecular patterns (DAMPs), including the dynamics of cell-free DNA (cfDNA) and mitochondrial DNA (mtDNA), endothelial injury/shedding markers, and metabolic patterns consistent with hypoxia–reperfusion stress (22). Such readouts can be used to define immune–metabolic endotypes that plausibly map onto injury nodes and thereby enable biomarker-guided enrichment in early-phase trials.

### 2.4 Remodeling risk as a downstream integrator

Remodeling is best treated as the integrated consequence of early network states. Rather than relying on late clinical events alone, a translationally efficient framework combines early mechanistic endpoints (necrosis, MVO/IMH, microcirculatory resistance, immune–metabolic trajectories) with intermediate remodeling surrogates [LV volumes, strain-based mechanics (23), natriuretic peptides (24)]. This layered endpoint design supports a clearer chain of inference: which compartment was modified, when, and whether that modification plausibly explains downstream remodeling, with cardiac magnetic resonance (CMR) serving as a central tool to track structure–tissue–function coupling and provide trial endpoints (25). Table 1 contrasts traditional single surrogates with the proposed network-aligned endpoint stacks.

**Table 1:** Traditional single surrogates vs. network-aligned endpoint stacks in reperfused AMI.

Clinical domain	Traditional surrogate (common)	Network-aligned endpoint stack (proposed)	Timing window (typical)	Dominant compartment mapped
Myocardial salvage	Infarct size (enzymes/CMR LGE)	(i) Necrosis (LGE/enzymes) + (ii) edema trajectory (T1/T2 mapping) + (iii) functional reserve (regional strain)	0–7 days (serial if possible)	Cardiomyocyte vulnerability
Tissue reperfusion	TIMI flow grade	(i) ST-segment resolution & myocardial blush grade; (ii) IMR (± CFR/RRR); (iii) CMR MVO (early/late) + IMH (T2*)	Minutes–days (CMR 2–7 days)	Microvascular bottleneck
Inflammation	Peak CRP/leukocyte count	(i) Resolution slope (*Δ* day1→day4/5); (ii) DAMP dynamics (cfDNA/mtDNA); (iii) endothelial injury/shedding markers; (iv) immunometabolic signatures (lactate/pyruvate; lipidomics if feasible)	Hours–week (serial)	Systemic immune–metabolic programming
Remodeling	LVEF at 3–6 months	(i) LV volumes+strain trajectory; (ii) natriuretic peptide trajectory; (iii) integrate acute MVO/IMH burden as risk modifier	Weeks–months	Downstream remodeling attractor state

## 3 The spatiotemporal injury network in myocardial I/R

Building on clinically anchored endpoints, myocardial I/R injury can be mapped as a spatiotemporal, multicompartment network in which actionable mechanisms change across minutes to weeks and across cardiomyocytes, microvasculature, and systemic immunity–metabolism. The aim of this section is not to restate classical pathways in isolation, but to emphasize mechanisms and transitions that reshape how targets and windows should be conceptualized and tested.

### 3.1 Time as an active variable

**Figure 1:**
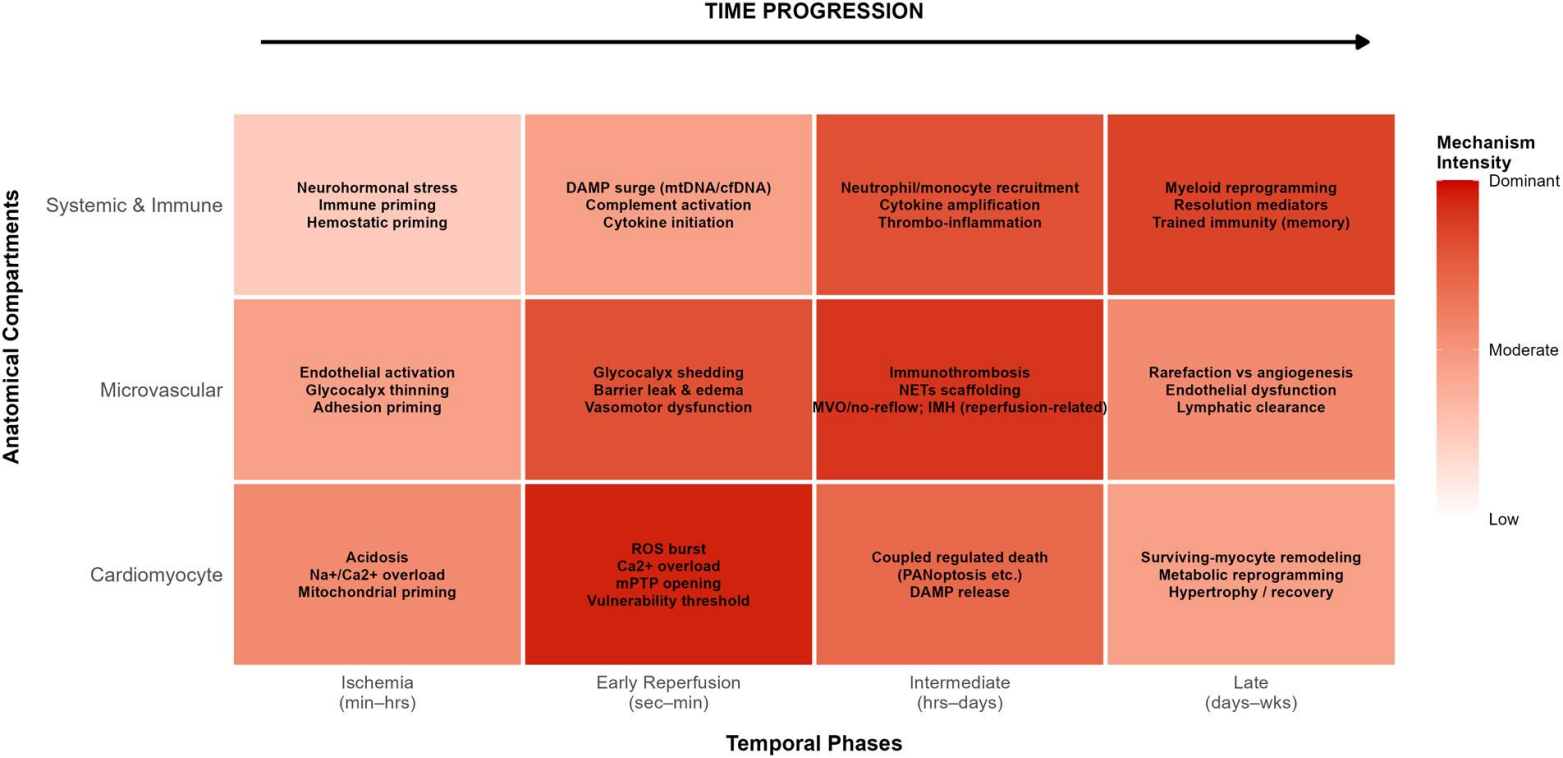
The spatiotemporal injury network in myocardial ischemia–reperfusion. The injury network is organized across four overlapping temporal phases—ischemia, early reperfusion, the intermediate phase, and the late phase—and three interacting compartments: cardiomyocytes, the coronary microvasculature, and systemic immunity–metabolism. Within each phase–compartment cell, dominant mechanistic modules are summarized, and color intensity indicates their relative prominence. Mechanism intensity reflects relative phase–compartment dominance and does not imply strict temporal separation or mutual exclusivity. DAMPs, damage-associated molecular patterns; IMH, intramyocardial hemorrhage; mPTP, mitochondrial permeability transition pore; MVO, microvascular obstruction; NETs, neutrophil extracellular traps.

A practical staging framework recognizes four overlapping phases: ischemic priming, early reperfusion triggering, intermediate propagation, and late remodeling. During ischemia, energy depletion, acidosis, ionic imbalance, and mitochondrial stress accumulate while many death programs become poised rather than fully executed. Early reperfusion introduces rapid pH normalization, oxygen reintroduction, calcium oscillations, oxidant bursts, and abrupt hemodynamic changes, while simultaneously accelerating sterile inflammatory signaling that can magnify injury (26). This period often sets an upper bound for salvage because mitochondrial vulnerability can convert into irreversible failure and microvascular barrier injury can be initiated (3). Over hours to days, regulated death execution, release of DAMPs, innate immune recruitment, endothelial activation, and immunothrombotic microvascular events reinforce one another, with reperfusion reshaping both the tempo and phenotype of immune-cell responses (27). Over days to weeks, resolution capacity, fibro-inflammatory remodeling, microvascular rarefaction or repair, and mechanical adaptation shape long-term ventricular structure and function. The four-phase staging framework is illustrated in Figure 1, and Table 2 summarizes dominant modules, objectives, and candidate readouts across these phases. These phases are presented for organizational clarity rather than as biologically discrete states, because cardiomyocyte, microvascular, and systemic processes overlap extensively *in vivo*.

**Table 2:** Spatiotemporal injury network: dominant modules, actionable objectives, and candidate readouts.

Phase	Dominant cardiomyocyte modules	Dominant microvascular modules	Dominant systemic modules	Actionable objective	Example readouts
Ischemia (min–h)	acidosis; Na^+^/Ca^2+^ loading; mitochondrial priming	glycocalyx thinning; endothelial activation	neurohormonal stress; myeloid priming	preserve “salvageable state”	ischemic time; collateral indices; early biomarkers
Early reperfusion (sec–min)	oxidant burst; Ca^2^+ oscillation; mitochondrial vulnerability threshold	glycocalyx shedding; barrier leak; shear-triggered dysfunction	early DAMP spillover	prevent threshold crossing; prevent barrier collapse	ST resolution; blush grade; early endothelial injury markers
Intermediate (h–d)	coupled regulated death (apoptosis/necroptosis/pyroptosis/ferroptosis)	immunothrombosis; NET scaffolding; microthrombi; edema/compression	innate recruitment; sterile inflammation amplification	limit propagation and compartment coupling	serial DAMPs; IMR; early CMR MVO
Late (d–wk)	debris clearance; remote-zone adaptation	repair vs. rarefaction; lymphatic clearance	resolution vs. trained-immunity–like memory	steer toward adaptive repair	CMR IMH/MVO evolution; LV remodeling; strain trajectory

Phases overlap substantially *in vivo* and are presented for organizational clarity rather than as discrete biological states.

### 3.2 Cardiomyocyte compartment: mitochondrial vulnerability and coupled death programs

Classical descriptions emphasize reactive oxygen species (ROS) and calcium overload converging on opening of the mitochondrial permeability transition pore (mPTP) (28). A network view generalizes this into a mitochondrial vulnerability state composed of interacting axes: redox instability, calcium mishandling, impaired mitochondrial dynamics, and insufficient quality control through mitophagy and proteostasis (29). Reperfusion tests this vulnerability state; if a threshold is crossed, cells transition from reversible dysfunction to irreversible failure. This framing shifts therapeutic design away from a single gatekeeper toward state stabilization, and it naturally accommodates human heterogeneity where the limiting axis differs among patients.

Mitochondrial dynamics and mitophagy are not merely housekeeping; they determine whether stressed mitochondria are repaired, isolated, or allowed to propagate dysfunction across the mitochondrial network. Excessive fission can fragment the reticulum and exacerbate dysfunction, whereas inadequate fission or mitophagy can prevent removal of damaged organelles. Importantly, increased mitophagy is not uniformly protective because timing and bioenergetic context determine whether quality control supports survival or contributes to energetic collapse (30). Phase-appropriate modulation that supports early stabilization and selective clearance without driving energetic insolvency is therefore the translationally relevant objective.

The cardiomyocyte death landscape in reperfused AMI includes apoptosis, necroptosis, pyroptosis, ferroptosis, and other regulated programs (31). Increasingly, the critical concept is that these programs are coupled through shared upstream stressors and mutual reinforcement via inflammation and the release of DAMPs. As a result, tissue injury can be robust to single-pathway blockade; when one executor is inhibited, the network may route injury through parallel executors. This coupling provides a plausible explanation for why single-pathway cardioprotection can fail despite apparent target engagement in simplified models and supports strategies that stabilize upstream vulnerability states or coordinate modulation of convergent inflammatory–death nodes.

Emerging frameworks should be incorporated with evidence-level restraint. PANoptosis is useful as an explanatory model because it formalizes co-regulation and partial co-execution among apoptosis, pyroptosis, and necroptosis, highlighting integration points that may reduce overall death-network tone (32). Copper-dependent metabolic-death proposals can be discussed as candidate modifiers of mitochondrial vulnerability rather than established dominant mechanisms in human reperfused AMI (33). The appropriate scientific posture is to link such concepts to testable predictions and tractable readouts, while clearly stating uncertainty.

A newer integrative layer emphasizes nucleic-acid danger signaling as a bridge between mitochondrial injury and sterile inflammation. Mitochondrial disruption can release mtDNA and related signals that act as potent inflammatory triggers, amplifying injury beyond initially damaged cardiomyocytes (34, 35). This perspective underscores that mitochondria are both energetic hubs and potential sources of inflammatory propagation, and it reinforces the logic that mitochondria-stabilizing therapies could reduce injury through dual mechanisms: preserving energy metabolism and limiting DAMP-driven amplification.

From a TCM-network perspective, emerging mechanisms such as PANoptosis and cuproptosis are best treated as integrative vulnerability modules rather than as validated standalone clinical targets in reperfused AMI. This is relevant because several TCM-derived compounds are reported to modulate convergent upstream processes—including oxidative stress, mitochondrial injury, inflammasome signaling, and proteostatic imbalance—that could, in principle, influence these death-integration nodes. However, for most candidates, current evidence remains limited to pathway-associated preclinical observations, and direct proof of human target engagement is lacking.

### 3.3 Microvascular compartment: barrier failure, immunothrombosis, and biomechanical bottlenecks

In reperfused AMI, microvascular dysfunction can develop despite restored epicardial flow (36). Endothelial activation, oxidant exposure, complement signaling, and leukocyte adhesion converge on barrier disruption. The endothelial glycocalyx supports mechanotransduction and permeability control, and its rapid degradation at reperfusion shifts the microvascular state toward leakiness, edema, and impaired vasoreactivity. Because these processes are temporally aligned with early reperfusion, microvascular injury can function as an independent determinant of tissue reperfusion quality rather than merely a downstream consequence of necrosis.

No-reflow and MVO increasingly align with an immunothrombotic mechanism in which platelets, neutrophils, monocytes, and coagulation signaling cooperate to produce microthrombi and inflammatory amplification (37). NETs can provide scaffolding for microthrombi and intensify endothelial dysfunction, contributing to the hybrid nature of microvascular obstruction (38). A direct translational implication is that intensifying anticoagulation may be insufficient if the dominant bottleneck involves NET scaffolding or barrier collapse, and escalation is constrained by bleeding risk in PPCI-era care. A more discriminating approach is to identify which immunothrombotic submodule dominates in a given patient population and match interventions to that submodule and time window.

Microvascular failure also has a mechanical dimension. Edema and swelling can physically compress capillaries; perivascular cells may contribute to sustained microvascular constriction; and local stiffness changes can impair capillary recruitment (11). These bottlenecks help explain persistent perfusion defects even when thrombotic signals are controlled. No-reflow is therefore often a multimodal bottleneck in which biochemical and biomechanical components vary in dominance across patients and time.

Beyond the acute obstruction event, microvascular recovery depends on repair and clearance systems, including lymphatic drainage of interstitial fluid and immune mediators (39). Inadequate clearance can prolong edema and inflammatory signaling, indirectly sustaining microvascular dysfunction and promoting maladaptive remodeling. This introduces a late-phase lever in which improving resolution infrastructure may not substantially change early necrosis but could shift recovery toward better function and less adverse remodeling.

### 3.4 Systemic and inter-organ compartment: immune–metabolic programming and injury memory

The post-reperfusion immune response is better described as state transitions than as a single peak. Metabolic intermediates can function as signals that shape immune cell phenotypes. Lactate is central in I/R because it reflects perfusion mismatch and metabolic stress; lactate-linked signaling and lactylation have been proposed as mechanisms influencing macrophage programming and the balance between inflammatory amplification and resolution (40). Current evidence in reperfused AMI remains evolving and context-dependent, and future work must clarify directionality, cell-type specificity, and causal relevance.

Reperfused AMI can mobilize immune cells from remote reservoirs and engage neuroimmune pathways, contributing to heterogeneity in inflammatory trajectories even with similar infarct sizes. A growing late-phase hypothesis is that AMI can imprint persistent inflammatory propensity through trained-immunity–like programs in innate immune cells (41), while stressed stromal or immune cells may develop senescence-like secretory phenotypes that perpetuate low-grade inflammation and fibrosis. Within a network framework, these processes function as memory nodes that stabilize maladaptive remodeling attractors.

Extracellular vesicles provide a plausible mechanism for transmitting stress signals (RNAs, proteins, lipids) across cell types and organs (42). Although causal chains and measurement standardization remain active areas, vesicle-mediated communication aligns with a network view in which injury propagation occurs not only through soluble cytokines and cell migration but also through packaged information transfer.

Lactate-linked signaling and lactylation are likewise better viewed as candidate regulators of immune–metabolic state transitions than as established clinical targets in reperfused AMI. For TCM-derived interventions, the key research gap is not whether a compound changes an isolated lactylation-associated marker *in vitro*, but whether it can reproducibly reshape post-reperfusion inflammatory-resolution trajectories *in vivo* and, ultimately, in clinically relevant human settings.

### 3.5 Integrative synthesis: endotypes, windows, and implications for therapy

**Figure 2:**
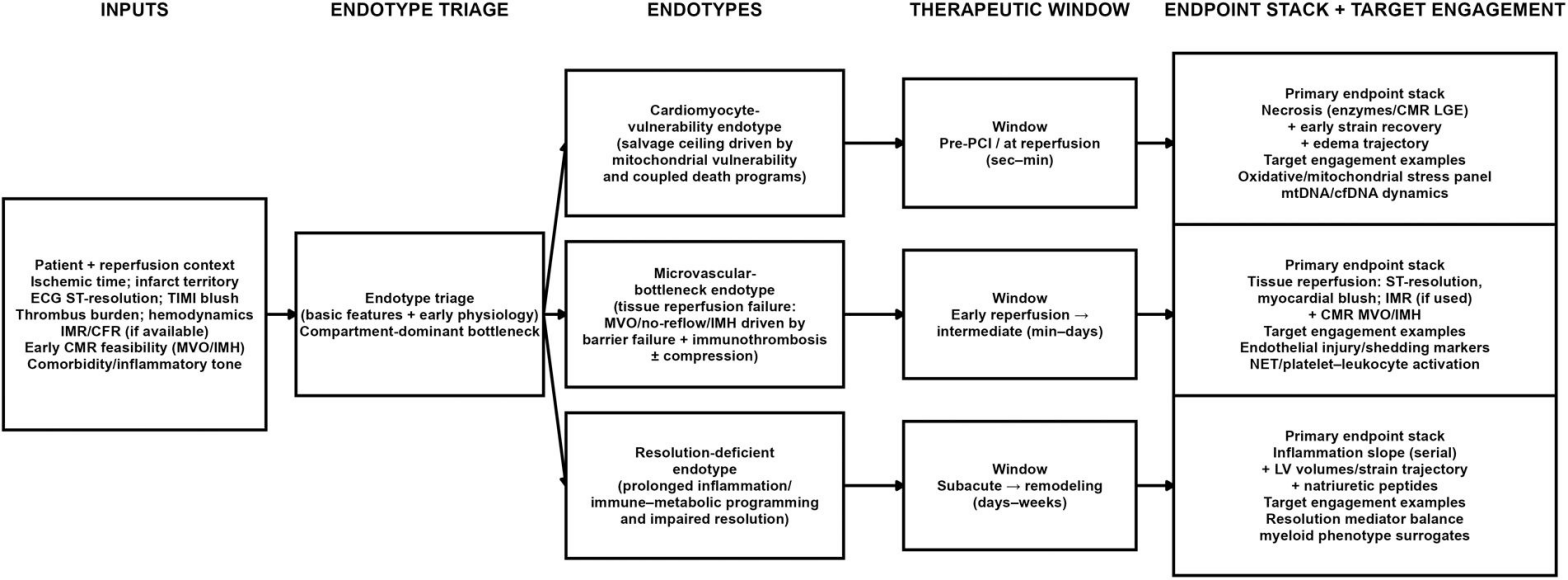
Endotype–window–endpoint selection workflow for reperfused acute myocardial infarction. This schematic illustrates a pragmatic framework for aligning therapeutic strategy with the dominant bottleneck after reperfusion. Patient and reperfusion context are used to triage patients into three endotypes: cardiomyocyte vulnerability, microvascular bottleneck, and resolution deficiency. For each endotype, the figure indicates the most plausible therapeutic window and a corresponding endpoint stack combining compartment-relevant efficacy measures with target-engagement biomarkers. The workflow is intended as a pragmatic enrichment-and-endpoint selection framework rather than a rigid disease sequence. CMR, cardiac magnetic resonance; IMH, intramyocardial hemorrhage; IMR, index of microcirculatory resistance; MVO, microvascular obstruction.

The spatiotemporal map suggests that reperfused AMI is better represented as a set of endotypes defined by dominant bottlenecks rather than a single uniform state (43). These endotypes are not strictly sequential or mutually exclusive stages. Rather, they represent overlapping dominant bottlenecks within the same reperfusion-injury network; more than one endotype may coexist in a given patient, while their relative dominance can shift over time. A cardiomyocyte-vulnerability endotype is characterized by early mitochondrial fragility and tightly coupled death programs that set an upper bound for salvage. A microvascular-bottleneck endotype is dominated by MVO/no-reflow and IMH driven by barrier failure, immunothrombosis, and/or biomechanical compression, thereby limiting tissue reperfusion despite epicardial patency. A resolution-deficient endotype is characterized by prolonged immune–metabolic programming and an impaired transition to resolution, which stabilizes adverse remodeling trajectories. These endotypes imply different therapeutic priorities and distinct endpoint sensitivities, motivating the alignment of interventions with phase-specific objectives using compartment-relevant endpoint stacks and target-engagement biomarkers (13). Table 3 summarizes trial-facing operationalization strategies, including practical enrichment signals and candidate readouts. A pragmatic endotype–window–endpoint selection workflow is summarized in Figure 2.

**Table 3:** Endotype framework for reperfused AMI: operationalization for trials.

Endotype	Operational “dominant bottleneck”	Enrichment flags (examples)	Primary objective by phase	Most sensitive endpoint stack	Target-engagement biomarkers (examples)
Cardiomyocyte-vulnerability	early mitochondrial fragility drives cell death and infarct expansion	short ischemic time; large AAR; high metabolic stress; early LV dysfunction disproportionate to MVO	early containment (minutes–hours)	necrosis+early strain recovery+edema dynamics	oxidative/lipid peroxidation signatures; mtDNA/cfDNA dynamics; mitochondrial stress-response panels
Microvascular-bottleneck	no-reflow/MVO/IMH limits tissue reperfusion despite epicardial patency	poor ST resolution; low blush grade; high thrombus burden; high IMR; early CMR MVO/IMH	barrier+immunothrombosis interruption (minutes–days)	IMR+CMR MVO/IMH+perfusion indices	endothelial shedding markers; NET-related markers; platelet–leukocyte activation panels
Resolution-deficient	prolonged inflammation and impaired resolution drive remodeling	comorbid burden; persistent inflammatory markers; early adverse edema/strain trajectory	accelerate resolution (days–weeks)	inflammation slope+strain/LV volume trajectory+natriuretic peptides	pro-resolving mediator balance; monocyte/macrophage phenotype surrogates; persistent DAMP signals

## 4 Why current therapies underperform: recurring translational barriers

Despite compelling mechanistic rationale and decades of experimental success, most cardioprotective strategies have failed to deliver consistent benefit in contemporary reperfused AMI. Neutral outcomes do not necessarily invalidate underlying biology; they often reflect a mismatch between intervention design and a shifting, endotype-dependent network (44).

Many candidate therapies were developed around a dominant-node hypothesis, assuming that inhibiting a single necessary event at reperfusion should yield substantial salvage. However, I/R injury exhibits rapid node switching and extensive pathway coupling. The same clinical phenotype may arise from different bottlenecks, and the same bottleneck may be driven by different submodules across patients. Consequently, single-node therapies face biological dilution through compensatory routing and population dilution because only a subset of enrolled patients exhibits the target-dominant endotype within the actionable window (13).

These barriers are illustrated by repeated neutral outcomes from large STEMI/PPCI trials of mechanistically plausible strategies. Pharmacologic inhibition of mitochondrial permeability transition with cyclosporine did not improve outcomes in PPCI-era settings (45). Remote ischemic conditioning similarly failed to improve major outcomes in a large contemporary trial context (46). Microvascular-targeted thrombolytic strategies have also yielded largely neutral signals in modern reperfusion care. Collectively, these experiences underscore that failure may reflect misalignment of endotypes, timing, and endpoints rather than irrelevance of I/R biology.

Endpoint selection further contributes to dilution. Reliance on infarct size or enzyme release implicitly treats myocardial salvage as the single most sensitive readout, yet microvascular and immune–metabolic dimensions often dominate recovery (47). Patients with substantial microvascular injury may have poor outcomes even when infarct size changes modestly, while cardiomyocyte-centered therapies may reduce necrosis without restoring tissue perfusion (48). Network-aligned trial design therefore favors endpoint stacks that include microvascular physiology or imaging indices, inflammatory-trajectory markers, and early functional readouts rather than a single surrogate (49).

Another recurring limitation is insufficient demonstration of target engagement in humans. In experimental models, dosing and timing can be controlled and tissue biomarkers can be measured directly. In clinical PPCI, dosing is constrained by logistics, safety, and concomitant medications, and tissue sampling is not routine. Neutral outcomes are therefore difficult to interpret without biomarker bridges that confirm exposure and engagement in the relevant compartment and time window (50).

Human heterogeneity and background therapies reshape the network. Age, diabetes, obesity, chronic kidney disease, and prior cardiovascular disease alter mitochondrial function, endothelial biology, and immune tone (51). Contemporary STEMI care includes potent antiplatelet agents, anticoagulation, statins, beta-blockers, and renin–angiotensin system inhibition, and evolving cardiometabolic pharmacotherapy may further modulate immunometabolic pathways (52). These factors can attenuate or mask effects observed in young, healthy animal models and reduce the probability that a single mechanism remains rate-limiting across a broad trial population.

Finally, compartment mismatch is underappreciated. Many cardioprotective agents were optimized around cardiomyocyte-centric mechanisms, yet microvascular dysfunction can dominate tissue reperfusion quality and downstream recovery (53). If no-reflow or MVO persists, cardiomyocyte rescue may not translate into functional recovery because oxygen delivery and metabolite clearance remain constrained. Conversely, microvascular-oriented therapies may improve perfusion and remodeling risk even with modest infarct-size effects, but such benefits can be missed if trials do not capture perfusion-sensitive endpoints.

**Figure 3:**
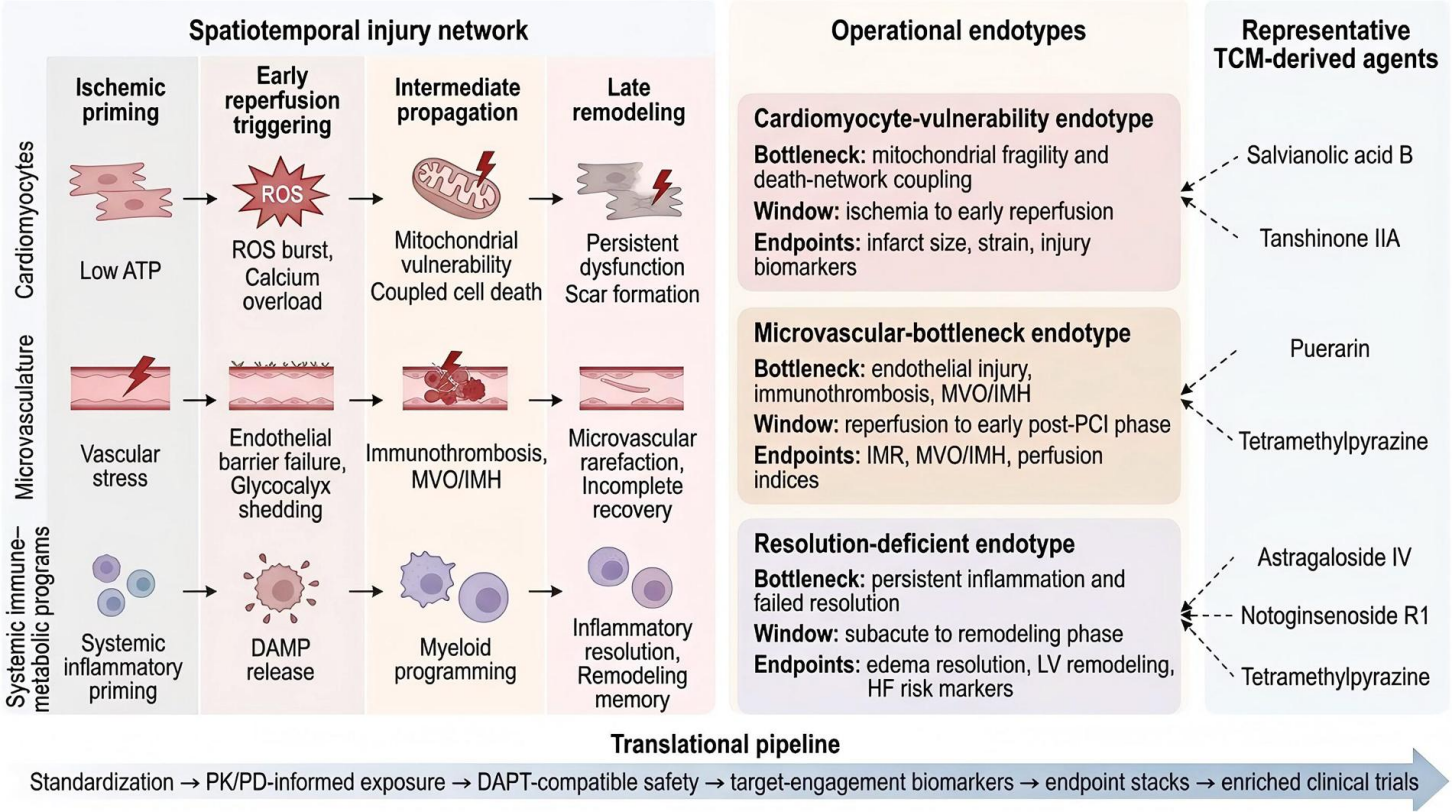
Integrative overview of endotypes, representative TCM-derived agents, and the translational pipeline in myocardial ischemia–reperfusion injury. The schematic integrates the conceptual framework developed in this review. The left panel summarizes the spatiotemporal, multicompartment organization of myocardial I/R injury across cardiomyocytes, the coronary microvasculature, and systemic immune–metabolic programs. The center panel presents three operational endotypes—cardiomyocyte vulnerability, microvascular bottleneck, and resolution deficiency—together with their dominant bottlenecks, therapeutic windows, and endpoint orientations. The right panel maps representative TCM-derived agents to the endotype domain to which they are most plausibly aligned for illustrative summary. The bottom panel outlines the translational pipeline for clinical development, including standardization, PK/PD-informed exposure, DAPT-compatible safety, target-engagement biomarkers, endpoint stacks, and enriched clinical trials. The figure is intended as an integrative conceptual summary rather than a rigid biological sequence or definitive therapeutic classification.

Taken together, these constraints suggest that progress requires explicit alignment across endotypes, windows, and endpoints. Interventions should be tested in cohorts enriched for a mechanistically matched bottleneck, delivered within the window where that bottleneck is actionable, and evaluated with endpoint stacks and target-engagement biomarkers that can detect compartment-specific effects (54). To facilitate rapid understanding of the overall framework developed in this review, Figure 3 provides an integrative summary linking the spatiotemporal myocardial I/R injury network, operational endotypes, representative TCM-derived agents, and the translational pipeline for endotype-matched clinical development.

## 5 Network-aligned multi-target modulation by defined TCM-derived agents and standardized preparations

A spatiotemporal network view reframes cardioprotection from identifying a single best target to designing interventions that modulate multiple, time-shifting bottlenecks across compartments. In this setting, defined bioactive compounds derived from traditional Chinese medicine and selected standardized preparations are of interest because many exhibit multi-node activity patterns that plausibly align with distributed I/R biology (55). However, multitarget activity is not intrinsically beneficial. To be credible in a PCI-era translational context, candidate agents should be evaluated through explicit node mapping, phase-appropriate timing logic, measurable target engagement, and compatibility with contemporary background therapy, particularly antithrombotic regimens.

A network-aligned review therefore differs from traditional pathway-based narratives. Instead of categorizing agents as “antioxidant” or “anti-inflammatory,” the emphasis shifts to identifying which bottleneck is most likely to be rate-limiting in a given endotype and whether an agent can plausibly reach, engage, and modulate that bottleneck within the relevant window. Evidence stratification is essential. Computational network inference and *in vitro* hypoxia/reoxygenation assays are hypothesis-generating; small-animal I/R outcomes provide proof of biological plausibility; endpoint-rich designs that include microvascular readouts improve interpretability; and human target-engagement data are necessary before large clinical outcome testing (56). Without this stratification, mechanistic novelty risks functioning as rhetoric rather than testable biology.

### 5.1 Representative defined compounds: node mapping, windows, and translational readouts

To avoid overinterpreting heterogeneous evidence, TCM-derived candidates discussed in this review are considered within a practical four-tier evidence-grading framework (Table 4), which distinguishes clinically interpretable candidates from translationally promising, exploratory, and concept-generating observations.

**Table 4:** Practical evidence-grading framework for TCM-derived candidates in myocardial I/R injury.

Translational grade	Core evidence basis	Typical supporting data	What this grade means	Major limitations before next-step translation
Grade A	Human mechanistic and/or early clinical evidence	Early-phase clinical studies; biomarker-defined target engagement; PCI-compatible safety data; preliminary PK/PD characterization; imaging or physiology-supported endpoint shifts	Clinically interpretable candidate with evidence beyond preclinical plausibility	Still requires confirmation of reproducibility, endotype enrichment, and outcome relevance in larger studies
Grade B	Robust and convergent preclinical evidence	Consistent findings across multiple *in vivo* I/R models and complementary *ex vivo*/*in vitro* studies; dose–response data; compartment-relevant endpoints (e.g., MVO/IMH, strain, inflammatory trajectories); mechanistic coherence across studies	Translationally promising candidate supported by more than single-pathway observations	Lacks direct human target engagement and PCI-era safety/interaction data
Grade C	Exploratory preclinical evidence	Single-model animal studies, limited replication, pathway-focused mechanistic data, or incomplete endpoint coverage	Hypothesis-generating candidate with biologically plausible but still preliminary support	Limited robustness, uncertain generalizability, and insufficient compartment-specific validation
Grade D	In silico or concept-level evidence only	Network pharmacology, molecular docking, enrichment analysis, or indirect pathway inference without convincing biological validation in myocardial I/R models	Concept-generating signal rather than a translational candidate	Requires experimental validation before mechanistic or clinical claims can be made

This framework is intended to guide translational interpretation rather than to provide a definitive ranking of efficacy. Grades reflect the maturity and interpretability of the evidence base, not intrinsic therapeutic superiority.

The compounds below are included as representative anchors for a node-mapped, evidence-aware approach rather than an exhaustive list.

Salvianolic acid B is frequently positioned as a compound that influences redox handling, mitochondrial stability, and inflammatory amplification. Within a network framework, its most plausible role is attenuation of mitochondrial–inflammation coupling during early reperfusion and the subsequent propagation phase (57). Translationally meaningful evaluation should include proximal biomarkers of oxidative and mitochondrial stress alongside time-resolved inflammatory and perfusion-related readouts, rather than relying exclusively on infarct size at a single time point. A major gap in much of the preclinical literature is limited exposure–response characterization and insufficient microvascular endpoint integration.

Tanshinone IIA and related derivatives illustrate a recurring challenge for multi-node candidates: reported mechanisms vary by model system, dose, and timing. In a spatiotemporal framework, context-dependence is plausible, but it increases the need for PK/PD anchoring and pre-specified target-engagement readouts (58). For translation, the priority is to define whether clinically feasible exposure can cover the intended window and whether engagement can be demonstrated using biomarkers that map to the proposed node modules.

Puerarin is commonly discussed in relation to stress-response regulators and inflammatory programs, with experimental studies implicating effects across autophagy-related and redox-linked pathways. In the network model, puerarin is most defensibly framed as a modulator of intermediate-phase amplification-vs.-resolution trajectories rather than a pure infarct-size reducer (59). Consequently, evaluation should prioritize trajectory-sensitive endpoints such as edema-resolution dynamics, early strain recovery, and serial immune–metabolic profiling.

Astragaloside IV is often linked to mitochondrial quality control and remodeling-relevant programs. Within a staged network, its most plausible window may extend beyond early reperfusion into the subacute and remodeling phases, where inflammation–fibrosis coupling and resolution infrastructure become rate-limiting (60). For remodeling-oriented candidates, a proof-of-concept objective may be improved resolution and remodeling trajectories rather than acute infarct-size reduction.

Notoginsenoside R1 and related Panax notoginseng saponins are frequently reported to influence stress-kinase signaling and inflammation-related nodes in I/R models. In a network-aligned framework, the key missing piece is compartment resolution: whether modulation primarily reduces cardiomyocyte death-program coupling, improves microvascular integrity, or reshapes systemic immune trajectories (61). Studies that integrate remodeling-sensitive readouts with microvascular and cardiomyocyte-proximal indices would further strengthen mechanistic mapping.

Tetramethylpyrazine (ligustrazine) is most plausibly aligned with the microvascular-bottleneck domain, given its frequent positioning as a microcirculation-facing candidate. If the intended clinical value is microvascular rescue, perfusion-first validation is essential, and PCI-era safety constraints must be treated as central rather than peripheral (62). In particular, any agent with plausible platelet or coagulation effects must be evaluated for bleeding risk and interactions with dual antiplatelet therapy and anticoagulation.

### 5.2 Standardized preparations as network modulators

Standardized preparations may offer translational advantages because they can be deployed within realistic clinical workflows and may achieve more reproducible exposure than loosely defined multi-herb interventions, provided that compositional control and manufacturing quality are rigorous (63). At the same time, ingredient complexity complicates attribution of target engagement, makes exposure–response relationships harder to define, and raises particular concerns regarding compatibility with contemporary antithrombotic therapy (64). Accordingly, standardized preparations should be evaluated not only as complex pharmacological products, but as clinically deployable network modulators whose composition, dosing logic, safety profile, and PCI-era feasibility all require explicit validation (65).

Safety evaluation should be operationalized rather than stated generically. In PCI-era AMI care, most patients receive dual antiplatelet therapy (DAPT), peri-procedural anticoagulation, statins, beta-blockers, and renin–angiotensin-system inhibition; therefore, any TCM-derived compound or standardized preparation with putative antiplatelet, anticoagulant, vasodilatory, cytochrome P450 (CYP)-modulating, or transporter-modulating activity should undergo structured interaction assessment before efficacy testing. A practical early-phase framework would include: (1) chemical standardization and batch consistency; (2) *in vitro* screening for CYP and transporter effects; (3) platelet-function and coagulation testing in the presence of aspirin and a P2Y12 inhibitor; (4) predefined bleeding surveillance, including Bleeding Academic Research Consortium (BARC)-classified events; and (5) pharmacokinetic/pharmacodynamic (PK/PD) analysis sufficient to determine whether concomitant acute coronary syndrome (ACS) therapy alters exposure or target coverage. This approach is especially important for preparations positioned as microcirculation-enhancing or anti-thrombotic adjuncts, because theoretical benefit at the microvascular level may be offset by excess bleeding or by interactions with contemporary DAPT regimens.

### 5.3 Moving beyond peak cytokines: trajectory reshaping and resolution capacity

Many TCM-derived candidates are described as anti-inflammatory, but the network view emphasizes that clinically meaningful benefit may derive from reshaping trajectories rather than suppressing peaks (66). Mechanistically aligned validation should incorporate serial immune phenotyping and DAMP-related signals that reflect the system's transition toward resolution (67). This approach can distinguish nonspecific suppression from improved resolution capacity and can explain why some interventions might reduce remodeling risk even when acute infarct size changes are modest.

### 5.4 Formulation, PK/PD, and translational constraints as mechanism-enabling variables

Mechanistic claims implicitly assume adequate exposure in the relevant compartment at the relevant time, which is rarely demonstrated. Formulation and delivery strategies should therefore be treated as integral to mechanism testing rather than optional enhancements. This is particularly important for early reperfusion targets where minutes matter and where microvascular obstruction may limit delivery to the very tissue that requires protection. Translationally, endpoint selection should be planned to capture both tissue-level injury and downstream functional consequences in a way that matches the hypothesized mechanism and time window (68).

### 5.5 Population modifiers and personalized deployment of TCM-derived interventions

A network-oriented TCM strategy should also account for population-level modifiers that reshape dominant injury bottlenecks. Patients with prolonged ischemic time may present with extensive irreversible necrosis and limited salvageable myocardium, reducing the likely value of purely mitochondria-centered rescue strategies. By contrast, patients with diabetes, obesity, chronic kidney disease, or advanced age may show greater endothelial dysfunction, altered immune tone, impaired microvascular recovery, and different pharmacokinetic exposure profiles, making microvascular-protective or resolution-oriented strategies more relevant. Accordingly, candidate TCM-derived interventions should not be discussed as universally applicable adjuncts, but rather as endotype-matched options whose use is conditioned by ischemic duration, comorbidity burden, bleeding risk, and background antithrombotic therapy.

## 6 Translational roadmap: endotype-matched, biomarker-anchored trial design

The core practical implication of a network framework is that translation should be structured around iterative learning rather than single-step outcome testing (69). Early-phase clinical studies should prioritize PCI-compatible feasibility, exposure, and safety, while simultaneously establishing human target engagement using pre-specified biomarker panels that map to the intended network nodes. Once engagement is demonstrated, efficacy testing should move to endotype-enriched cohorts with endpoint stacks aligned to the dominant bottleneck and window, consistent with precision-oriented thinking in ischemia–reperfusion injury more broadly (70).

Endotype enrichment can be approached pragmatically. For microvascular-bottleneck phenotypes, enrichment may rely on early angiographic and electrocardiographic (ECG) markers of poor tissue reperfusion, invasive microcirculatory indices when available, and early imaging where feasible. For cardiomyocyte-vulnerability phenotypes, enrichment is more likely when salvageable myocardium remains substantial, and when early functional impairment is disproportionate to microvascular injury. For resolution-deficient phenotypes, enrichment may emphasize comorbidity burden and early trajectory markers suggesting persistent inflammation or delayed recovery.

Across all endotypes, trial interpretability improves when endpoints are layered: proximal target-engagement biomarkers provide mechanistic evidence that the intended node is modulated, while intermediate imaging, physiology, and functional readouts reveal whether modulation shifts the network state in the predicted direction. This structure supports rational go/no-go decisions before large outcome trials and reduces the probability of late-stage failure without mechanistic learning, aligning with broader arguments that multifactorial injury often requires multitarget strategies rather than single-node suppression (71).

## 7 Discussion and conclusions

Myocardial I/R injury after AMI in the PPCI era is best treated as a spatiotemporal network in which cardiomyocyte death programs, microvascular perfusion failure, and systemic immune–metabolic programming jointly determine recovery and remodeling. The repeated underperformance of single-target approaches is consistent with node switching, pathway coupling, and endotype heterogeneity rather than absence of biology. A therapy can be mechanistically relevant yet clinically neutral if it is delivered outside the dominant therapeutic window, targets a compartment that is not rate-limiting for tissue recovery, or exerts distributed effects that are not captured by the selected endpoints.

This perspective clarifies how TCM-derived compounds and standardized preparations should be evaluated. Their potential advantage is not nonspecific breadth but coordinated partial modulation of multiple bottlenecks, provided that such modulation is demonstrable, phase-appropriate, and compatible with contemporary background therapy. Translational credibility requires rigorous standardization, PK/PD-informed dosing, explicit evaluation of PCI-era safety and interactions, and biomarker-guided trial designs enriched for mechanistically matched endotypes. Under these conditions, multi-node modulation becomes quantifiable and optimizable rather than a general claim.

We do not argue that multitarget activity is inherently superior to single-node intervention. Rather, in a network-structured injury state such as reperfused AMI, partial modulation of several coupled bottlenecks may be more biologically realistic than attempting to suppress a single pathway across a heterogeneous clinical population. This hypothesis, however, requires rigorous standardization, human target-engagement evidence, and PCI-compatible safety testing before clinical claims can be made.
